# Analysis of the Reasons for the Tearing of Strips of High-Strength Electrical Steels in Tandem Cold Rolling

**DOI:** 10.3390/ma14237124

**Published:** 2021-11-23

**Authors:** Ivan Petryshynets, František Kováč, Ladislav Falat

**Affiliations:** Institute of Materials Research, Slovak Academy of Sciences, Watsonova 47, 04001 Košice, Slovakia; fkovac@saske.sk (F.K.); lfalat@saske.sk (L.F.)

**Keywords:** high-strength silicon steels, crystallographic texture, cold rolling, brittle damages, 5-stand tandem cold rolling mill

## Abstract

High-strength non-oriented electro-technical steels with a low thickness possess excellent isotropy of electromagnetic and mechanical properties which is highly required in the production of high-efficiency electric motors. The manufacturing process of this type of steel includes very important and technologically complex routes such as hot rolling, cold rolling, temper rolling, or final heat treatment. The final thickness is responsible for the decrease in eddy-current losses and is effectively achieved during cold rolling by the tandem rolling mill. Industrial production of thin sheets of high-strength silicon steels in high-speed tandem rolling mills is a rather demanding technological operation due to the increased material brittleness that is mainly caused by the intensive solid solution and deformation strengthening processes, making the dislocation motion more complex. The main objective of this work was to investigate the distribution of local mechanical strains through the thickness of high silicon steel hot bands, generated during the cold rolling. The experimental samples were analysed by means of electron back-scattered diffraction and scanning electron microscopy. From the performed analyses, the correlation between the material workability and the nucleation of cracks causing the observed steel strip failure during the tandem cold rolling was characterized. Specifically, the microstructural, textural, misorientation, and fractographic analyses clearly show that the investigated hot band was characterized by a bimodal distribution of ferrite grains and the formation of intergranular cracks took place only between the grains with recrystallized and deformed structures.

## 1. Introduction

Fe-Si electrical steels play an important role in the generation and distribution of electricity [1] as well as in electrical appliances and devices on the consumer side [2]. The worldwide activities are directed to the improvement and optimization of the steel grades in order to fulfil direct requirements for the energy efficient and reliable performance of the electrical machines [3]. Variations of silicon steels grades differ with respect to their magnetic properties, sheet thickness, mechanical properties, the value of electrical resistivity, and the type of coating. In the last decades, there has been a strong demand for the development of super high-speed traction motors used in hybrid electric and fully electric vehicles [4]. The development of new electrical motors requires that the rotor of the motor operate at very high rotary speeds, i.e., rounds per minute (RPM). The design conditions for this situation of very high rotation require that the steel components of the rotor in the form of electrical steel laminations should possess a sufficiently high yield strength to resist the resulting high centrifugal forces. The requirement of high yield strength is the additional essential pre-condition for the electrical steel material used in considered heavy-duty electro-motors as the ordinary material requirements rely on the selection of the steel grades fulfilling only the electro-magnetic properties. The steels used in hybrid motors should be based on the grades with low thickness and high resistivity, achieved through high alloying of the steel, primarily with silicon and aluminium, in order to assure low electrical losses at high frequencies. The requirement for high yield strength comes in addition to the requirement for low electrical losses. Thus, it is necessary that the rotor and stator core materials possess both the excellent magnetic properties and required mechanical strength in order to sustain high centrifugal forces acting on the motor [5,6,7,8].

The electro-magnetic characteristics of “fully-finished” grades of non-oriented (NO) electrical steels are extremely dependent on the microstructure and on the desired crystallographic texture components of rotated cube {001}<110> or Goss {110}<001>. The existing crystallographic texture in fully recrystallized ferrite materials determines the magnetic induction behaviour at high values and frequencies of the applied external magnetic field. The microstructural parameters strongly affect the hysteresis and eddy-current losses [9,10,11]. To obtain a suitable texture and microstructure, precise control of the process is necessary during all the individual manufacturing steps. This involves adjusting the chemical elemental composition during the steel making, tuning the appropriate temperature and reduction control during hot rolling, controlling the temperature during hot band annealing, controlling the temperature and deformation during cold rolling, and controlling the heating rate and temperature during final annealing [12,13,14].

The electro-magnetic characteristics, such as electrical resistance, magnetic induction, and permeability are improved as the content of silicon increases [15,16,17,18,19]. Specifically, by increasing the Si content the electrical resistance also increases, the eddy current and hysteresis losses decrease (especially at high frequencies), and the magnetization becomes almost zero. Therefore, the silicon is added up to 4.5% in high strength NO electro-technical steel sheets. The saturation magnetization decreases with an increase in the amount of Si because Si is a non-magnetic element. For this reason, it is difficult to produce a material which possesses high magnetic flux density and low iron loss only by controlling the amount of Si added [20,21,22,23,24,25]. Furthermore, by increasing the silicon content, solid solution strengthening of the electro-technical steel strips occurs, which causes the embrittlement of the material. Therefore, it is difficult to perform cold rolling to obtain thin sheets, especially by employing high-speed tandem rolling mills (TRM) [26,27,28,29]. Thus, in industrial operational practice, unexpected tearing (i.e., fracturing) of high-silicon steel strips during the cold-rolling process is always a serious issue for various steelworks. As already mentioned, by increasing the Si content, the strength characteristics and hardness of the material increase. On the other hand, due to silicon-induced material embrittlement, the deformation properties significantly decrease, which results in possible difficulties in the rolling processes of high-silicon electrical steels [30,31,32].

Prior to the cold-rolling process, high-grade NO silicon steel is required to undergo a heat treatment procedure. The strip annealing after the hot rolling can be performed by two methods, either by hot band annealing in a continuous annealing line or the normalized annealing of coils, depending on the technological possibilities. In the continuous process, the annealing temperature is in the range from 1050 °C to 1100 °C with a holding time up to 60 s. In the case of the coils’ annealing, the annealing temperature is lower and lies in the range from 750 °C to 900 °C with a holding time of 3 h. This annealing step induces an important re-orientation of the microstructure (including the development of the texture of the material) which results in significantly lower losses and a higher permeability for high-grade electrical steels [33,34]. The application of this procedure has a positive effect on the magnetic properties of the final strips, but due to the heat dissipation during cooling, the temperature of a part of the head portion and the tail portion of the strip steel is often somewhat lower than that of its middle portion. As a result, the rolling stability is poor, and fractures may accidentally occur during cold rolling in TRM. It is important to note that in the case of high-strength electrical steel, both the hot strip annealing, and technological parameters of the cold rolling process are critical. The effects from the inhomogeneity of the microstructure throughout the hot strip thickness also play a significant role with respect to the strain hardening, local strain distribution, and nucleation of failures [35,36,37,38]. It is well-known that the microstructures of hot strips of electrical steels with Si content above 3 wt.% are characterized by a bimodal morphology. In accordance with the Hall–Petch relation [39], an inhomogeneous level of strain hardening occurs in individual micro-areas. Fedorov et al. [40] employed the dislocation pile-up model in a description of crack nucleation at the internal grain boundary near to a free surface of a metal with a bimodal grain size structure.

In recent years, a lot of work has focused on improving the microstructural, textural, and mechanical properties of NO electrical steels. He and Hilinski [41] investigated the unconventional cold rolling scheme realized by the reverse cold rolling mill. They reported that steels with 2.8% Si were rolled down to 0.5 mm without mechanical damage. Hayakawa et al. [42] used the cross-rolling methods for cold rolling electrical steels with 3.3% Si. They showed that experimental steels were characterized by the desired deformability during the rolling and an improved crystallographic texture after the final annealing process. Jiao et al. [43] investigated the innovative warm rolling process on a single roll rolling mill which could replace cold rolling for high strength NO electrical steels. All these mentioned methods of rolling in comparison with 5-stand rolling mills are characterized by a very long rolling process. That is why the mentioned innovative rolling techniques are not suitable for application in industrial conditions.

The aim of our present work is the analysis of the effects of microstructural and textural characteristics of high-strength electrical steel hot band on its tearing during high-speed tandem cold rolling.

## 2. Materials and Methods

The experimental material studied in this work was a fractured segment of commercially produced vacuum degassed hot bands from the production line of high-strength silicon NO electrical steel of M250-50A grade. The chemical composition of the experimental material determined by perchloric acid dehydration gravimetric method is shown in Table 1.

**Figure 1:**
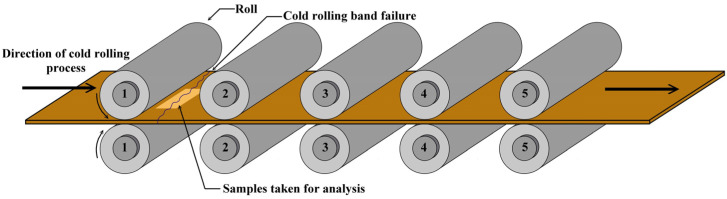
Scheme of a 5-stand tandem cold rolling mill with the failure location of the experimental hot band.

Hot rolled strips after coiling and hot band annealing were used as referential experimental samples. The thickness of these samples was 1.8 mm. The next samples were taken from the experimental hot band after its rupture during the cold rolling by a 5-stand tandem cold rolling mill. In the tandem cold rolling process (see Figure 1), a metal strip is passed through five pairs of independently driven work rolls. As the strip passes through the individual pairs of work rolls, the thickness is successively reduced by very high compression stress in a small region (i.e., the roll bite) between the work rolls. The necessary compression force is applied by hydraulic rams, or by a screw arrangement driven by an electric motor [44]. Our experimental hot band was fractured between the first and second work roll as shown in Figure 1. In order to investigate the reason for the tearing of the high strength silicon hot band, the samples were taken from the vicinity of the failure edge of the band, see Figure 1. The thickness of these samples was 1.38 mm.

**Figure 2:**
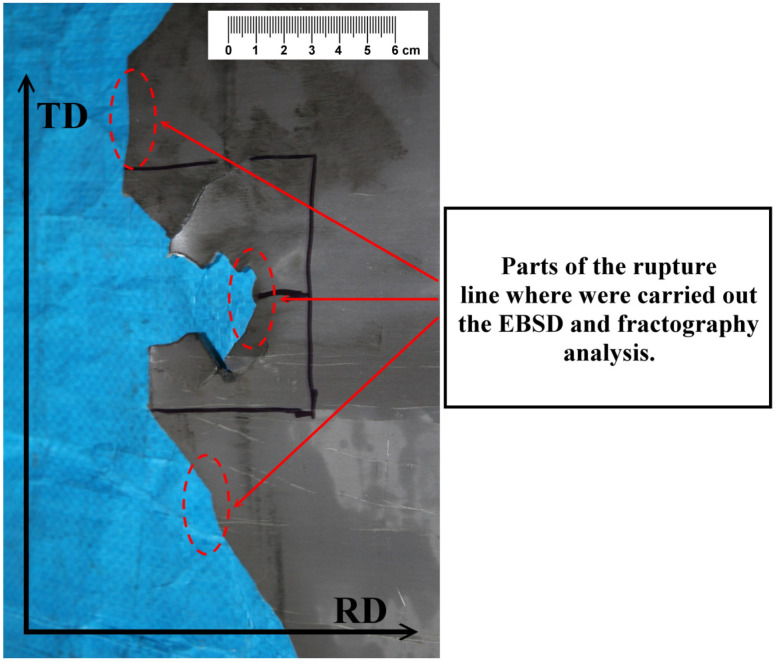
The failure edge of the fractured hot band.

The part of the ruptured strip is presented in Figure 2. As can be seen, the failure edge shows a non-linear morphology; however, it is mostly perpendicularly oriented to the rolling direction of the hot band. This part of the fractured strip was used for the analysis of the failure edge. The samples from the marked area in Figure 2 were used for complex failure analysis including the microstructural, fractographic, and crystallographic texture analyses. The samples for the analyses were cut by electrical discharge machining using a spark erosion machine EIR-EMO 2N (Emotek s.r.o., Nové Mesto nad Váhom, Slovakia).

Microstructural analyses of the selected investigated samples corresponding to individual material states were carried out using a light optical microscope (LOM) OLYMPUS GX71 (OLYMPUS Europa Holding GmbH, Hamburg, Germany). In order to obtain a statistical distribution of the grain size, the selected microstructural states were processed by using the free metallographic analysis software ImageJ. This enabled an estimation of the average grain size in the different parts of the microstructure of the samples.

Room-temperature tensile tests were carried out using a universal testing machine TIRATEST 2300 (TIRA GmbH, Schalkau, Germany) at a strain rate of 0.11 s^−1^ in order to investigate the effects of tensile deformation on the crack nucleation mechanism in the studied hot band microstructure. Tensile tests were performed in order to estimate the yield stress and the ultimate tensile strength of our investigated hot band. The mechanical test was carried out on three specimens in the rolling and the transverse directions. The mechanical properties of the studied samples are presented in Table 2.

The fractographic and crystallographic texture analyses were performed by employing a scanning electron microscope (SEM) JEOL JSM-7000F (Jeol Ltd., Tokyo, Japan) equipped with electron backscattered diffraction (EBSD) detector Nordlys-I (HKL technology A/S, Hobro, Denmark). The texture analyses were carried out using the EBSD method in the normal direction plane for each sample of 25 mm × 10 mm in size. The recorded EBSD data were processed by the CHANNEL-5, HKL software package (Service pack 7).

Tescan Vega-3 LMU (TESCAN Brno, s.r.o., Brno, Czech Republic) was used for the selected samples taken from the edge of the ruptured hot strip specimens in order to investigate their fracture surface.

The metallographic preparation of the microstructure of the samples was carried out by grinding and polishing. Firstly, specimens were grinded consistently by following silicon carbide grinding papers with grit 180, 320, 500, 800, 1200, and 2400. All samples were finished by grinding paper with grit 4000 and then were polished by diamond paste with grain sizes of 1µm and 0.25 µm. Surface etching was performed in Nital solution. In the case of EBSD analysis, the microstructure polishing was finished by colloidal silica suspension with a grain size of 0.04 µm.

## 3. Results and Discussion

### 3.1. Microstructure of the Hot Band

**Figure 3:**
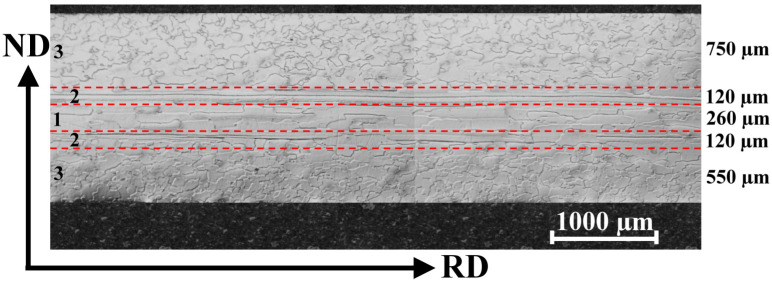
The initial (as-received) microstructure of the investigated hot band strip.

The initial microstructure of the investigated steel obtained from the industrial line in the form of a hot band is illustrated in Figure 3. The light-optical microstructure observations were carried out on metallographic cross-sections of individually selected samples. As one can see, their longitudinal part is oriented parallel to the rolling direction. The microstructure analysis indicates that the sample is characterized by a bimodal distribution of ferrite grains with different sizes and shapes across the sample thickness.

Three different types of grain morphologies in various parts of the hot band cross-section can be distinguished. The middle part of the experimental sample is characterized by sizeable polygonal grains elongated along the rolling direction. Moreover, these grains of the central region can be further differentiated into the fully recrystallized grains and highly elongated non-recrystallized grains.

The first type of grains with a fine uniaxial morphology are located in the central region of the sample, i.e., area 1 in Figure 3. The performed image analyses indicated their average dimensions to be about 1000 µm ± 100 µm along the RD and about 150 µm ± 12 µm along the ND. The highly elongated grain structures can be observed in area 2 that is lying on both neighbouring sides of area 1. The lengths of these grains along the RD are about 5–10 mm. Finally, the subsurface regions (i.e., area 3) of the investigated sample are characterized by migrated grain boundaries with an irregular zig-zag morphology. The average size of these subsurface grains is around 150 µm ± 12 µm. The thickness of the observed areas is specified on the right part of the microstructure in Figure 3.

In the present case, the hot band annealing was used in order to soften the material by the effects of the recovery and recrystallization processes. Schneider et al. [8] have claimed that the evolution of the microstructure during the hot band fabrication depends critically on the finishing hot rolling parameters, such as the annealing temperature, time, and heating rate of the coiling of the hot bands during the box annealing prior to the cold rolling. As already shown in Figure 3, the microstructure of the investigated sample is rather inhomogeneous across the thickness, i.e., partly recrystallized subsurface regions with irregular-shaped grains coexist with still deformed regions, especially in the central part. On the basis of our observation, it can be concluded that some of the used processing parameters of either hot rolling (i.e., reduction degree, temperature) or those of the box annealing heat treatment were not suitable for achieving a homogenous microstructure in the investigated hot bands.

### 3.2. Texture and Local Misorientation Profile

**Figure 4:**
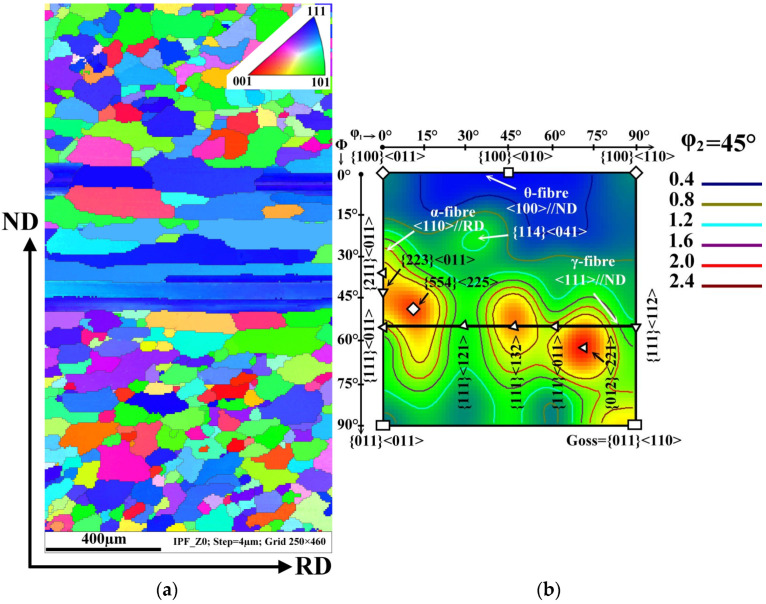
Characterization of the crystallographic orientation of the experimental hot band in the initial state: IPF map (**a**) and ODF section taken at φ_2_ = 45° (**b**). The colour key for the identification of crystallographic orientation of grain is located in the upper right corner of the IPF map.

The crystallographic texture is an important parameter influencing not only the magnetic properties of NO electrical steels in the final state but also the plasticity and workability of hot bands during the final cold rolling process. In order to characterize the texture evolution of the investigated hot band before and after the damage in the 5-stand tandem rolling mill, specific EBSD analyses were performed. The common texture of the experimental steel in its initial hot band after box annealing is shown in Figure 4. The crystallographic orientation of the matrix grains across the cross-section of hot band is presented by an Inverse Pole Figure (IPF) map and the orientation distribution function (ODF) in Figure 4a,b, respectively. The IPF visualization (Figure 4a) of the microstructure provides a complementary crystallographic characterization to the light-optical microstructure observation (Figure 3). The performed EBSD analyses demonstrate that the irregularly-shaped grains located in the subsurface region are characterized by a Goss texture (green grains) or a rotated cube {001}<110> (red grains) texture. The longitudinal non-recrystallized grains and the fine uniaxial grains in the central part of the investigated cross-section are represented mostly by the blue colour tone which is responsible for the strong deformation texture {111}<uvw>, see Figure 4.

The most relevant texture components of the studied sample are α-, γ-, and θ-fibre represented by ODF in Figure 4b. As one can see, the initial microstructure of the hot band corresponds to three strong peaks at {554}<225>, {111}<132>, and {012}<221> a typical orientation on/or near γ- fibre, which represents the deformation texture <111>//ND. This sample also has a characteristic maximum at {011}<110> which is responsible for the strong Goss texture component. The cube crystallographic component is described by the weak intensities of the {100}<011> and {100}<110> textures which are on the θ-fibre at φ_1_ = 0° and φ_1_ = 90°, respectively. The EBSD measurements clearly show that the investigated hot band sample is characterized by a complicated morphology of grains showing an expressive gradient of their resulting crystallographic texture across the thickness.

**Figure 5:**
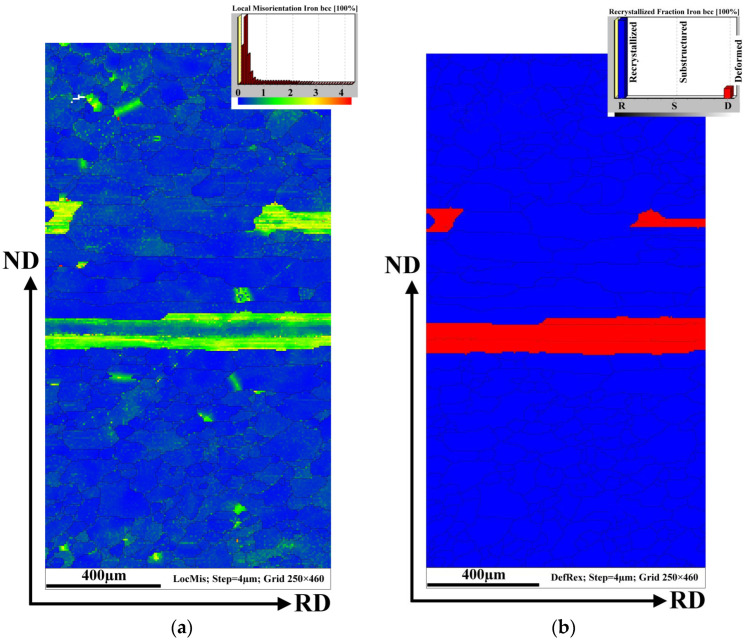
The local misorientation map of the microstructure of the initial sample with the legend (the upper right corner) for angles ranging from 0 to 5° which are marked by the rainbow colour and showing the higher deformation region (**a**), EBSD recrystallization fraction map (**b**).

Figure 5a represents the average local misorientation map (LMM) obtained from the EBSD data. It illustrates where there is a high level of misorientation within the hot band sheet. The high misorientation is an indication of the areas of mechanical strains which are related to plastic deformation. In other words, the value of local misorientation is related to the degree of deformation. Once the cold rolling reduction is imposed, the grains are forced to deform, which leads to the formation of dislocations. The accumulation of a high dislocation density produces high misorientation in strongly deformed regions. This type of analysis (Figure 5a) allows to us to identify the large amount of plastic mechanical strains which are visualized in the microstructure around or inside investigated ferrite grains. Here, the emphasis is given to resolving the substructural boundaries that are typically misoriented by more than 2°. The legend (upon the right corner) shows the intensity of the misorientation angles on the analysed surface. It is clearly visible that the major portion (blue tone colours) of the grain matrix is characterized by a very low misorientation angle (around 0°). This indicates that these ferrite grains were not plastically deformed. Nevertheless, the micrograph in Figure 5a also shows some grains with a high level of misorientation angles in the range of 2–5°. This indicates that these grains are characterized by an increased intensity of mechanical strain associated with a high dislocation density. Evidently, the deformed grains have a strongly elongated morphology in area 2 of the hot band microstructure cross-section (as shown in Figure 3). The fact that the elongated grains contain mechanical deformation is also confirmed by the recrystallization map in Figure 5b. In this case, the map illustrates only the recrystallized (blue) and deformed (red) fractions.

These areas were identified according to the misorientations of individual grains (see Figure 5a). In this sense, it can be seen that the recrystallized grain fraction is located in both the subsurface and mid-thickness regions which correspond to areas 3 and 2 in Figure 3. The deformed grains on this map are indicated by elongated grains located in area 2. It is evident that grains with a low misorientation angle are in the recrystallized material state which can be related to the fact that the investigated hot band strips were sampled after the box annealing. Despite the application of the annealing procedure, the elongated grains were found to be highly deformed.

**Figure 6:**
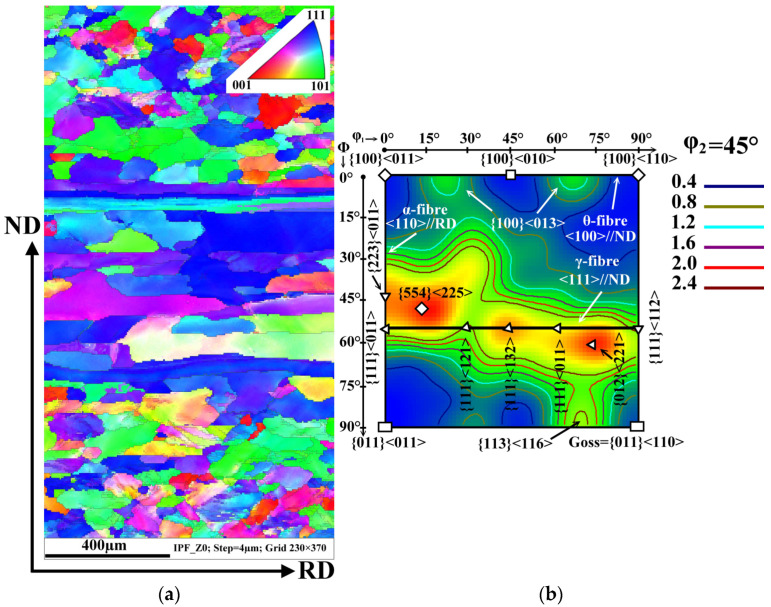
Characterization of the crystallographic orientation of the experimental hot band in a ruptured state: IPF map (**a**) and ODF section taken at φ_2_ = 45° (**b**). The key for the identification of the crystallographic orientation of the grain is located in the upper right corner of the IPF map.

The analysis of the microstructure and the crystallographic texture of the samples taken from the tearing strip edge (Figure 2) is presented in Figure 6. It is important to note that the thickness of these investigated samples was 1.38 mm, unlike the samples taken from the initial hot band with a thickness of 1.8 mm. Thus, it can be assumed that the material near the rupture line was subjected to about 23% of mechanical deformation. Figure 6a shows the microstructure and crystallographic texture of the investigated steel near the fracture surface.

The IPF map demonstrates that this part of the hot band is characterized also by inhomogeneous mixture of irregularly shaped zigzag grains in the subsurface region and the combination of elongated and sizeable uniaxial grains in its central part. The average grain size is similar as in the sample presented in Figure 3. The coloured map illustrates that the grains with a strong intensity deformation texture {111}<uvw> (blue colour) mostly covered the central part of the investigated sample. The ODF section shows that this specimen with a 23% reduction displays a strong γ-fibre texture. From comparison of IPF and ODF sections, it can be concluded that very elongated, deformed, and sizeable uniaxial recrystallized grains are mainly composed of strong {554}<225>, {111}<132> and {012}<221> texture components. The grain structure of the subsurface region is characterized mostly by the same weak {100} and {110} components such as {100}<013> and {113}<116> in combination with a low volume fraction of grains with a γ-fibre texture. The comparison of the experimental samples in the initial state and the ruptured state clearly indicates that their microstructure and crystallographic texture characteristics are similar.

**Figure 7:**
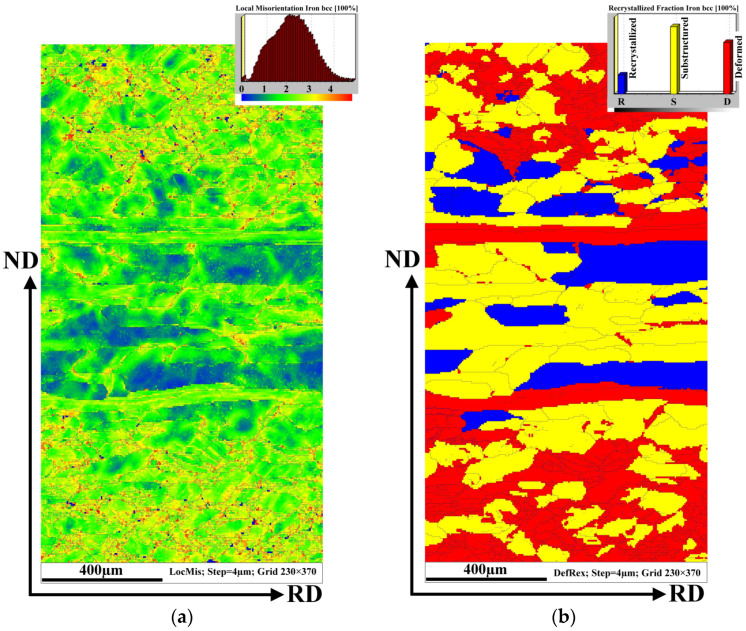
The local misorientation map of the rupture sample microstructure with the legend (the upper right corner) for angles ranging from 0 to 5° which are marked by the rainbow colour and showing the higher deformation region (**a**), EBSD recrystallization fraction map (**b**).

The behaviour of mechanical strains through the complicated inhomogeneous microstructure of tearing samples is presented in Figure 7. To further investigate the correlation between the grain boundary evolution and strain accumulation, the local misorientation mapping (LMM) for all EBSD maps was measured. Figure 7a illustrates the small orientation changes within each grain and between neighbouring grains, highlighting the regions of higher plastic mechanical stress. The value of local misorientation is mostly in the range from 0 to 5° (Figure 7a). The value of local misorientation is related to the degree of deformation which was applied to the hot strip during the rolling by the first roll on tandem rolling mill. As can be seen, the misorientation angles are distributed inhomogeneously throughout the observed microstructure. The higher intensity of the grain structures with the misorientation angles of about 5° are mostly observed in the subsurface region located between the sample surface and very elongated deformed grains. The central part of the samples showing recrystallized uniaxial grains is characterized by a low intensity of structural heterogeneities. In this case, the misorientation angle in the range of 3–5° was detected mostly in the vicinity of the grain boundaries.

The complex state of the grain matrix in the analysed sample is shown in Figure 7b. This coloured recrystallized map shows the recrystallized, substructured, and deformed fraction. Here, approximately 50% of the microstructure is formed of substructure grains. The substructure and recrystallized grains are located mostly in the central part of the cross-section. This fact corresponds with the results obtained from the local misorientation maps which illustrate that the sizeable uniaxial grains are characterized by a low degree of misorientation. It is possible to conclude that the grain matrix in the central part of the sample after the applied 23% of deformation is characterized mostly by undeformed recrystallized or weakly deformed substructural grains. On the other hand, the deformed grains are detected mostly in the subsurface region which in this case is limited by the deformed elongated grains.

A comparison of Figure 7a,b clearly demonstrates that the uniaxial grain subsurface matrix including the elongated grain structure absorbed the overwhelming majority of deformation induced by cold rolling reduction.

Taking into account the fact that the value of local misorientation is proportional to the degree of dislocation density, it should be noted that higher deformation occurred mostly near the grain boundaries where the irregular grain structures were also observed. Moreover, plastic mechanical strains induced by the accumulation of dislocations were mostly distributed in up to fine elongated grain structures which were detected in both initial and ruptured hot band samples. It can be clearly seen that the cross-section of the investigated ruptured sample has two zones of plastic mechanical strains. One of them was formed between the surface of the sample and elongated hereditary grain structures and is characterized by a high dislocation density mostly in the vicinity of the irregular zig-zag grain boundaries as well as inside the elongated deformed grains. The lowest value of plastic deformation was detected in the central part of the sample thickness, and it appeared mostly on the boundaries of uniaxial grains which are in the recrystallized or substructured state.

From the comparison of the microstrustural, textural, and misorientation results, it is possible to conclude that the mechanical plastic strain gradient was created through the cross-section of the hot band during the cold rolling reduction. The performed EBSD misorientation measurements indicate that the motion of dislocations was slowed down on the elongated grains which can be related to the dislocation pile-up effects. This behaviour is known to be especially pronounced in the case of the body-centered cubic (bcc) crystal structure and it is related to the so-called deformation crystallographic orientation in combination with coarse longitudinal grains [45,46].

### 3.3. Microstructure and Texture of Specimens Subjected to a Tensile Test

The influence of grain boundary structures on intergranular crack nucleation were analysed on the interface of recrystallized and deformed grain structures. These structures are shown in areas 3 and 2 in Figure 3. It is well known that the crack nucleation occurs mainly along grain boundaries and depends strongly on both the grain boundary character and grain boundary configuration with respect to the persistent slip bands. However, it is less dependent on the geometrical arrangements between the grain boundary plane and the stress axis. In particular, random boundaries become preferential sites for the crack nucleation [47].

**Figure 8:**
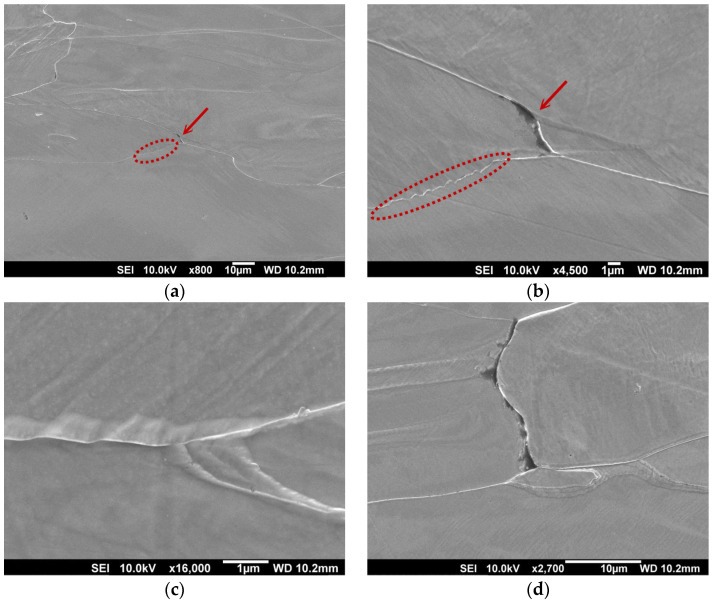
Microstructure in the longitudinal direction in the central zone of a partially deformed specimen (**a**), nucleation of persistent slips bands on the grain boundaries (marked by a dashed line) and a crack at the grain boundary (**b**), deformation zone around the boundary (**c**), decohesion at the boundary of two grains (**d**).

In order to analyse the role of morphological and crystallographic anisotropy of the microstructure, a tensile test was performed on a specimen from the ruptured part of the hot band, oriented transversally to the rolling direction. The tensile test was interrupted at the elongation of 15%. The investigation of such deformed material was carried out by the SEM-EBSD technique. The SEM micrographs of the grain-boundary crack nucleation stage in the sample which was taken from the tensile test specimen just before its rupture by mechanical testing are presented in Figure 8. These microstructures were obtained in the longitudinal direction in the central zone of the hot strip thickness. At the grain boundaries, the nucleation of the deformation steps and subsequently of the micro-cracks can be seen. Our analysis clearly shows that the cracks predominantly nucleated at grain boundaries and triple junctions, see Figure 8a–c. On the other hand, the crack nucleation along the persistent slip band in the grain interior was only observed in the vicinity of grain boundaries and triple junctions [48]. It should be noted that no nucleation of the cracks inside the grains or around the secondary inclusions was observed in the partially deformed sample. The deformation zones around the grain boundary and triple junction are presented in Figure 8b,c, respectively. The presented results indicate that the grain boundaries and the triple junctions were preferential sites for crack nucleation in the investigated material, which was characterized by the gradient of grain size, boundaries structures, crystallographic textures, and local mechanical strains.

**Figure 9:**
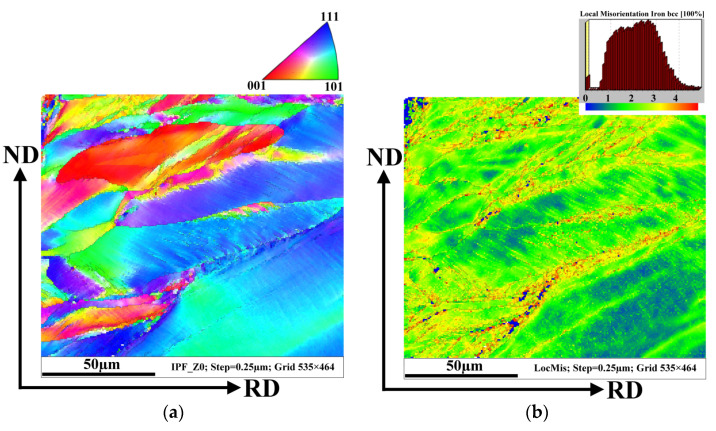
Typical EBSD dataset obtained during the SEM-EBSD test: IPF map representing the crystallographic texture of the grain vicinity in the deformation zone (**a**), (LMM) local misorientation map representing the intensity of mechanical strains vicinity of grain boundaries (**b**).

Analysis of the local mechanical strains in the vicinity of the grain boundaries in the sample used for the tensile test was carried out by the EBSD technique. Inverse pole figure (IPF) maps and LMM maps are shown in Figure 9a,b, respectively. Both misorientation and texture maps illustrate the increase in the tensile damage as the local misorientation and orientation change increased inside the grains. Especially in this highly strained sample, gradual orientation changes can be observed in the IPF map between the centres of the grains and the grain boundaries; this is especially notable in large grains. Each grain is defined as the area surrounded by boundaries with a misorientation above 5°. However, randomly oriented grains may have any degree of misorientation with respect to each other, and the fraction of the grains with a misorientation of less than 5° can be calculated mathematically [49,50].

The LMM values characterize changes in the local misorientation caused by geometrically necessary dislocations. Local misorientations plotted as a map represent the areas with an increased density of the defects. Regarding the dataset shown in Figure 9b, the LMM map for the strengthening material shows strong significant gradients inside the elongated grains, showing that the central part of these grains was practically strain-free. In this case, increased LMM values can be observed the along grain boundaries, indicating the formation of fine channels or slip lines at different locations. At the obtained strains, the locations of high intensity of misorientation in the deformed samples existed at the grain boundary intersections. In accordance with [51], their existence is indicative of increased local dislocation density and dislocation pile-up formation (see Figure 9b). The high LMM values were observed along the dislocation lines, suggesting the presence of dislocations inside the deformed zone. However, some grains with rough structures may have had a very small, if any, increase in the LMM value within a grain body, far from the grain boundary.

### 3.4. Fractographic Analysis

When the hot band is broken during the cold rolling reduction, the observed failure occurrence can be due to various reasons such as material property, degree of stress which caused the rupture, repeated effect of stress increase and decrease, temperature, time, and environmental conditions, etc. The cause of the rupture can also be revealed by a fractographic analyses of the fracture surfaces of broken samples enabling the identification of the failure mode and the fracture micro-mechanism.

**Figure 10:**
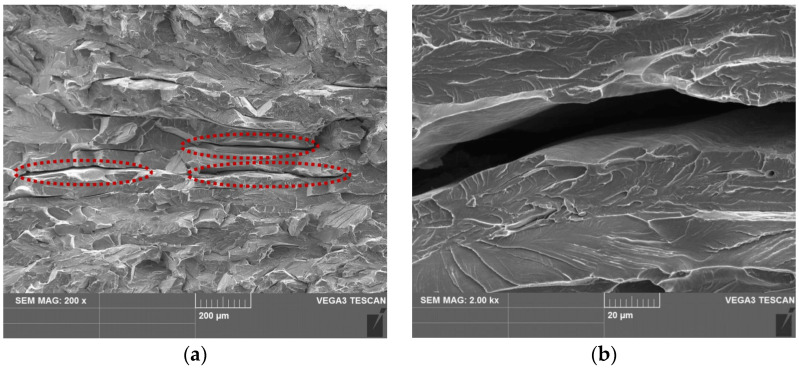
Fracture surface after the rupture of a specimen oriented in the transverse direction (**a**), detailed view on crack (**b**).

Figure 10 shows the fracture surface of the edge of the ruptured hot strip specimen (Figure 2). The SEM-fractographs illustrate the fracture surfaces on the cross-section of broken band which was oriented in the transverse direction (TD). The observed area was oriented in the rolling direction (RD). It can be seen that the fracture surface of the investigated sample exhibits highly inhomogeneous morphology from its upper edge (strip surface) towards central part of the cross-section. The similar distribution of inhomogeneous features was detected in the case of the microstructure (Figure 3) and texture (Figure 4, Figure 5 and Figure 6). Detailed observations clearly show that the subsurface areas are characterized by a transgranular cleavage fracture showing typical cleavage facets with river patterns. This fracture micro-mechanism is related to the occurrence of migrated grain boundaries with a zig-zag morphology which covered the subsurface region. In contrast, the central part of the sample cross-section with an elongated grain structures and sizeable uniaxial grains (Figure 3) is characterized by intergranular decohesion. The detailed observation of crack nucleation in Figure 8b shows that the mechanical fracture of the strip occurred mainly along the grain boundaries.

The performed failure analysis clearly indicates that intergranular cracks were formed only in the central part of the sample cross-section on the boundaries between the grains with a zig-zag morphology and elongated shapes as well as between the grains with elongated and uniaxial shapes. This observation supports our conclusions from the above discussed analyses correlating the texture and local misorientation measurements. All the performed analyses give rise to the conclusion that the mechanical rupture of the hot band was governed by the mechanical stress gradient in the vicinity of grains with an elongated morphology. It can be stated that near the elongated hereditary grain structures, mechanical cracks were nucleated and propagated which gradually led to plasticity exhaustion of the material induced by the pile-ups effect. An intercrystalline brittle fracture occurs when the deformation stress reaches the cohesive strength of the boundary. The cohesive strength is significantly reduced by the deformation steps at the boundary as a result of the dislocation motion within the grain [52]. The susceptibility to a brittle intercrystalline fracture is promoted by the tensile stress state, a high strain rate, a low temperature, a coarse grain structure, and textural heterogeneity [53]. Our analyses indicated a significant participation of coarse grain structure and textural heterogeneity to cause the observed tearing of strips of high-strength electrical steels in tandem cold rolling. Based on our analyses, it can be concluded that avoiding the studied failure of high-strength silicon steel might be possible by improving the homogeneity of the microstructure and the texture of the hot band by optimising the hot rolling process parameters. This approach will be the subject of our subsequent studies.

## 4. Conclusions

The aim of present study was to investigate the main reasons for the brittle failure of high-strength electro-technical steel during the cold rolling in a high-speed tandem rolling mill. The main conclusions can be summarized as follows:

- The microstructure of investigated hot band was characterized by a highly inhomogeneous morphology and size of grain structures across the band thickness. It was found that the microstructure was not fully recrystallized and the highly deformed regions were still presented in its central part. The results pointed to the fact that the parameters of hot rolling deformation or those of the box annealing heat treatment were likely not suitable for achieving the homogenous recrystallized microstructure throughout the whole thickness of hot bands.
- The analysis of crystallographic orientation has clearly shown that experimental hot strips exhibited the gradient crystallographic texture with strong intensity deformation texture {111}<uvw> in its middle part.
- The misorientation measurements through the cross-section of the ruptured hot band revealed two zones of plastic mechanical strains. One of them was formed between the surface of the sample and elongated hereditary grain structures and was characterized by high-intensity dislocation density, mostly in the vicinity of the zig-zag irregular grain boundaries as well as in the inside of elongated deformed grains. The lowest value of plastic deformation was detected in the central part of the sample thickness.
- The fractographic analysis of crack nucleation has clearly shown that the formation of intergranular cracks takes place only in the central part of the sample cross-section on the boundaries between the grains with a zig-zag morphology and elongated deformed structures.
- By comparing performed microstructural, textural, misorientation, and fractographic analyses it can be concluded that the mechanical plastic strain gradient was created through the hot band cross-section during the cold rolling reduction. Consequently, the motion of dislocations was slowed down on the elongated grains due to the dislocation pile-up effects leading to the plasticity exhaustion. The gradual formation of mechanical cracks inevitably resulted in observed tearing of strips of high-strength electrical steel during high-speed tandem cold rolling.

## Figures and Tables

**Table 1:** Chemical composition of the experimental hot strip material (wt.%).

Si	C	Mn	Al	P	Fe	Other Elements
3.21	0.006	0.25	0.18	0.04	97.95	<0.094

**Table 2:** The yield stress and ultimate tensile strength of the experimental hot bands.

	Rp0.2, [MPa]	Rm, [MPa]
Rolling direction	475 ± 11	595 ± 15
Transverse direction	503 ± 13	639 ± 14

## Data Availability

Not applicable.
